# Efficacy and safety of combination therapies vs monotherapy of hypomethylating agents in accelerated or blast phase of Philadelphia negative myeloproliferative neoplasms: a systematic review and meta-analysis

**DOI:** 10.1080/07853890.2022.2164611

**Published:** 2023-01-16

**Authors:** Jia Chen, Kefei Wang, Zhijian Xiao, Zefeng Xu

**Affiliations:** a State Key Laboratory of Experimental Hematology, National Clinical Research Center for Blood Diseases, Haihe Laboratory of Cell Ecosystem, Institute of Hematology and Blood Diseases Hospital, Chinese Academy of Medical Sciences & Peking Union Medical College, Tianjin, China; b Tianjin Institutes of Health Science, Tianjin, China; c MDS and MPN Centre, Institute of Hematology and Blood Diseases Hospital, Chinese Academy of Medical Sciences & Peking Union Medical College, Tianjin, China

**Keywords:** Myeloproliferative neoplasms, accelerated/blast phase, hypomethylating agents, ruxolitinib, venetoclax

## Abstract

**Background:**

There is a lack of evidence regarding whether combination therapy of hypomethylating agents (HMAs) has better outcomes than HMA monotherapy in patients with Philadelphia chromosome-negative accelerated or blast phase myeloproliferative neoplasms (MPN-AP/BP).

**Materials and methods:**

Pubmed, Embase, Web of Science and Cochrane library databases were searched for studies from inception of each database until 31 December 2021. Data extraction and synthesis were conducted following the PRISMA reporting guideline.

**Results:**

It was found that HMAs plus venetoclax therapy yielded a higher CR/CRi rate than HMAs alone [36% vs 19%, *p* = .0204] and a higher CR rate than HMAs plus ruxolitinib [22% vs 8%, *p* = .0313]. HMAs plus ruxolitinib combination showed a higher ORR than HMA monotherapy [45% vs 30%, *p* = .0395], but there was no improvement in CR/CRi. The one-year and two-year OS rate for patients treated with HMAs plus venetoclx/ruxolitinib demonstrated a trend towards prolonged survival than HMAs alone [HMAs plus venetoclax: 24% vs 11%, *p* = .1295 and 12% vs 3%, *p* = .2357; HMAs plus ruxolitinib: 25% vs 11%, *p* = .0774 and 33% vs 3%, *p* = .051].

**Conclusion:**

It was confirmed that HMA in combination with venetoclax is an effective and well-tolerated option in MPN-AP/BP patients in pre- as well as post-haematopoietic stem cell transplantation settings. HMA plus ruxolitinib therapy was revealed to be effective in patients with MPN-AP.

**Key Messages**
- Combination therapy with HMAs and venetoclax/ruxolitinib was associated with improved outcomes than HMAs alone in MPN-AP/BP patients.
- Further large-scale randomized controlled trials are needed to confirm regarding to the optimal treatment for this patient population.

## Introduction

The classical categories of Philadelphia chromosome-negative myeloproliferative neoplasms (MPNs) as listed in the 2016 World Health Organization (WHO) classification comprise a spectrum of disorders including primary myelofibrosis, essential thrombocythaemia and polycythaemia vera [1]. In general, the natural pathological process of MPNs can be interrupted by disease transformation into the accelerated or blast phase (MPN-AP/BP) [2,3]. MPN-AP and MPN-BP (or post-MPN acute myeloid leukaemia [post-MPN AML]) are defined based on the percentage of blasts in the bone marrow or peripheral blood, which is ≥10–19% in MPN-AP and 20% or more in MPN-BP [4]. MPN-AP/BP are portentous events and are likely emblematic of the late stages of the evolutionary history of MPNs.

At present, allogeneic haematopoietic stem cell transplantation (allo-HSCT) is the sole treatment modality with long-term survival data [5,6]. However, allo-HSCT is strongly discouraged in patients with MPN-AP/BP due to the poor treatment outcome [7]. Moreover, allo-HSCT is not an optimal option for many patients owing to their advanced age, poor performance status, comorbidities, inability to achieve a reasonable response, or lack of a suitable donor [6,7].

Less intensive regimens, such as, hypomethylating agents (HMAs, including azacytidine and decitabine) combined with or without venetoclax/ruxolitinib, may be effective for post-MPN AML patients [8,9]. HMA-based combination therapy (HMAs plus venetoclax/ ruxolitinib) has significant anti-tumour activity and is being increasingly recognized as an alternative to AML-like therapy for unfit MPN-AP/BP patients, with the exception of higher-risk myelodysplastic syndrome (MDS) and AML [10,11]. Rampal et al. demonstrated an overall response rate (ORR) of 53% and a median overall survival (OS) of 7.9 months in MPN-AP/BP patients treated with combined decitabine and ruxolitinib [12]. A multicentre retrospective study evaluated treatment outcomes of HMAs combined with venetoclax therapy among 32 MPN-BP patients [13]. Complete remission (CR) or CR with incomplete count recovery (CRi) was achieved in 44% of patients, and the median OS was higher compared to controls treated with HMAs alone (8 vs. 5.5 months), but the difference was not statistically significant (*p* = .3) [13]. However, due to the retrospective nature of the above reports and the limited number of participants, these findings cannot be considered conclusive. To our knowledge, no meta-analysis has been performed to compare the efficacy and safety of combination therapy vs monotherapy of HMAs in MPN-AP/BP patients.

The aim of this meta-analysis was to compare the efficacy and safety of HMAs combined with ruxolitinib or venetoclax to HMAs alone in patients with MPN-AP/BP.

## Methods

### Literature search strategy

We conducted a literature search for publications written in any language, by screening the Pubmed, Web of Science, Embase and Cochrane library databases, following a literature search strategy of keywords and Boolean operators presented in the Supplement. We also searched abstracts from the American Society of Hematology conferences, American Society of Clinical Oncology, European Hematology Association and European Society of Medical Oncology. A systematic search was performed from inception of each database until 31 December 2021. After removal of duplicates, two authors (J.C. and K.F.W.) independently screened all abstracts and titles against eligibility and exclusion criteria. Thereafter both reviewers assessed the full text of potentially relevant articles and decided whether or not to include them in the meta-analysis. The objectives and methods were predefined in a protocol registered at PROSPERO (CRD42022313523). This study followed the principles outlined in the Declaration of Helsinki and was conducted in accordance with the PRISMA (Preferred Reporting Items for Systematic Review and Meta-analyses) guidelines [14].

### Selection of studies and data extraction

Studies were selected based on the following inclusion criteria: (1) clinical trials and retrospective studies; (2) patients with BCR-ABL negative MPN-AP/BP, or post-MPN AML (no sex or age restrictions); (3) patients treated with HMA monotherapy or combined HMA with ruxolitinib/venetoclax; (4) providing outcome measurements, such as ORR, CR, CRi, partial response (PR), median OS or adverse events (AEs). All selected clinical trials were registered online. Two authors (J.C. and K.F.W.) independently extracted data following a standard format. Any disagreement was resolved through discussion with a third reviewer (Z.F.X.). Extracted information included study characteristics, patient characteristics, interventions, survival data and AEs.

### Quality assessment and bias risk

The results of quality assessment and bias risk are presented in the Supplemental information. The Methodological Index for Non-Randomized Studies (MINORS) scale [15] was used to evaluated the quality of all evidence (Supplemental Figure 1) and a heat map (Supplemental Figure 2) was plotted by using the R programming software. According to the MINORS scale, eight methodologic items (1–8) were assigned to the noncomparative studies, and four items (9–12) were designated for the comparative studies. These items were scored as 0 (not reported), 1 (reported but inadequate) or 2 (reported and adequate). Publication bias of studies (more than 10 studies) was evaluated by symmetry of the funnel plot and Egger’s test (Supplemental Figure 3).

### Definition of response and endpoint

The primary outcome of interest was the ORR, defined as a composite of CR, CRi and PR. Given the wide range of publication dates of the included studies and the lack of validated response criteria for MPN-AP/BP [16], definitions used by the individual publications varied slightly but were primarily based on the revised Cheson criteria [17]. Key secondary endpoint included OS and rate of allo-HSCT.

### Statistical analysis

The meta-analysis was conducted by using a common (fixed)-effects model according to the assumption of the similar effect size among studies, and the random-effects model was constructed to achieve consistency. All extracted outcome data underwent logarithmic transformation prior to pooling by inverse variance weighting method to improve the reliability of the combined results. As most of the selected studies were single-arm tests, we calculated the single ratio and then the integrated ratio with 95% confidence interval (95% CI) using the R package meta. Statistical heterogeneity between summary results was determined using Cochran’s Q test and I^2^ indices. The summary odds ratio (OR) and 95% CIs were calculated from the studies that contained data for the control group. Furthermore, we performed the planned subgroup analysis and univariate meta regression analysis to compare effect measurements of different studies based on the type of intervention. Sensitivity analysis was performed for the significant heterogeneity (defined as I^2^>60%; ≥3 studies) by using leave-one-out method. All analyses were performed using the R statistical software, version 4.1.2.

## Results

### Literature search

**Figure 1.**
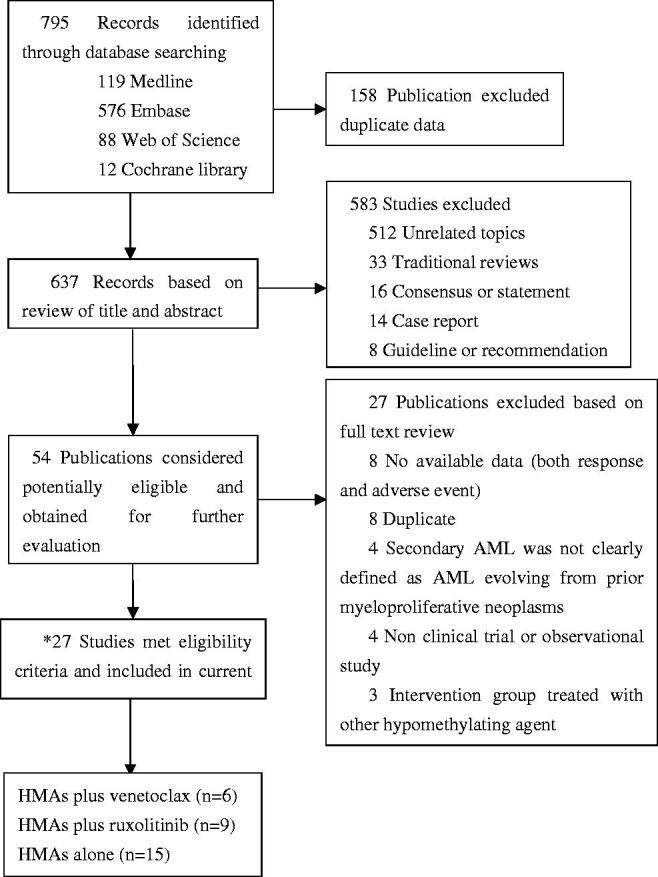
Flow diagram for selection of studies included in the meta-analysis. *Three studies (Lancman et al. [22] USA, 2018; Yoon et al. [29] USA, 2021; Zhou et al. [10] USA, 2021) reported the results of combination therapy as well as monotherapy of HMAs simultaneously.

Our literature search strategy identified 637 publications after removal of the duplicates. Based on title and abstract reviews, studies reporting results on disorders other than accelerated or blast phase of MPN or post-MPN AML, review articles, use of HMAs in combination with drugs other than ruxolitinib and venetoclax, case reports, and those presenting insufficient data (less than five patients) were excluded from the analysis. Finally, 27 studies were included in the qualitative synthesis (3 studies are included both in Tables 1 and 2 because they reported the results of combination therapy as well as monotherapy with HMAs). Figure 1 depicts the flow diagram outlining the study selection process. The general characteristics of the patients included in the individual studies selected for this meta-analysis are provided in Table 1 and Table 2.

**Table 1.** Overview of the efficacy and safety of combination therapy with HMAs in Philadelphia negative MPN-AP/BP.

Source (author/ country)	Study type	Population	Number of men, n (%)	Median age (year, range)	Regimen	Number of cycles, median(range)	Efficacy	Median OS, Months (range)	Adverse events
Bose et al. [18] USA, 2016	Clinical trial	*N* = 12 Post-MPN AML	8(66.7)	70(32–92)	DEC plus RUX	4(1–7)	ORR:5/12, 42%; CR/CRi: 3/12,25%; PR:2/12, 16.7%	NA	Grade ≥3: pneumonia, febrile neutropenia, thrombocytopenia, neutropenia, infection, hypotension, fracture, fever, fatigue, anorexia, muscle weakness, confusion, ileus and transaminitis
Rampal et al. [19] USA (Phase I), 2016	Clinical trial	*N* = 21 MPN-AP 11 MPN-BP 10	NA	63(48–88)	DEC plus RUX	3(1–22)	ORR:12/21, 57.1%; CR/CRi:7/21, 33%; PR: 5/21, 24%	10.4(3.3–NR)	Grade ≥3: infection, anaemia, leukopenia, thrombocytopenia, haemorrhage
Rita Assi et al. [20] USA, 2017	Clinical trial	*N* = 19 Post-MPN AML	11(58)	71(32–92)	DEC plus RUX	2(1–7)	ORR: 4/14, 29%; CR/CRi: 4/14, 29%	8(1–20)	Grade ≥3: febrile neutropenia, myelosuppression, fracture, fatigue, anorexia, muscle weakness, confusion, ileus and transaminitis
Drummond et al. [21] UK, 2017	Clinical trial	*N* = 14 MPN-AP: 7 MPN-BP: 7	NA	72(62–81)	AZA plus RUX	3(0–7)	ORR:2/6, 33%, CR:1/6, 16.7%; PR: 1/6, 16.7%	NA	Grade 1/2: constipation, vomiting, anaemia, nausea, abdominal pain, injection site reaction, dyspnoea, fever and hyperkalaemia; Grade ≥3: anaemia, febrile neutropenia, neutropenia, abdominal pain, hypotension, infections, thrombocytopenia, pneumonitis and syncope
Rampal et al. [12] USA, 2018	Clinical trial	*N* = 21 MPN-AP: 8 MPN-BP: 13	13(61.9)	63(48–88)	DEC plus RUX	3(1–34)	ORR:9/17, 53%; CRi:4/17, 23.5%; PR:5/17, 29.4%	7.9(4.1–NR)	Grade ≥3 haematologic AEs: neutropenia (33.3%), lymphopenia (19%), thrombocytopenia (19%), anaemia (14%); Grade ≥3 nonhaematologic AEs: febrile neutropenia (33.3%), pneumonia (29%), sepsis (14%), respiratory failure (9.5%), hypertension (9.5%), cellulitis (9.5%), gastrointestinal bleeding (9.5%), squamous cell carcinoma of the skin (9.5%)
Lancman et al. [22] USA, 2018	Retrospective study	*N* = 57 MPN-BP	33(58)	68(40–92)	DEC +/- RUX	2.5(1–32)	HMAs with or without additional therapies: CR: 9/57, 15.8%;	6.7	NA
Bose et al. [23] USA, 2020	Clinical trial	*N* = 29 Post-MPN AML	NA	69(32–92)	DEC plus RUX	2(1–8)	ORR:13/29,45%; CR:12/29,41.4%; PR:1/29, 3.4%	6.9(0.4–42.6)	Grade 1/2: nausea, pruritus, fatigue, diarrhoea, dizziness
Gangat et al. [24] USA, 2020	Retrospective study	*N* = 12 MPN-BP	6(50)	71(48–81)	DEC/AZA plus Venetoclax	3(1–5)	ORR:5/12, 42%, CR:3/12, 25%, PR:2/12, 17%	6(2–NR)	NA
Mascarenhas et al. [25] USA, 2020	Clinical trial	*N* = 25 MPN-AP:10 MPN-BP:15	11 (44)	71.0 (46.7–85.6)	DEC plus RUX	4(2–11)	ORR:11/25, 44%, CRi:2/25, 8.0%; PR: 9/25, 36%	9.5(4.3–12)	Grade ≥3 haematologic AEs: neutropenia (16%), anaemia 4 (16%) and thrombocytopenia (8%); Grade ≥3 nonhaematologic AEs: febrile neutropenia (28%), pneumonia (24%), bone pain (8%).
Tremblay et al. [26] USA, 2020	Retrospective study	*N* = 9 MPN-AP:1 MPN-BP:8	7 (77.8)	69 (49–72)	DEC/AZA plus Venetoclax	NA	ORR:3/9, 33.3%; CR/CRi:3/9, 33.3%,	4.2(2.5–NR)	Grade ≥3: infections, intracranial haemorrhage
Gangat et al. [13] USA, 2021	Retrospective study	*N* = 32 MPN-BP: 32	19 (59)	69 (47–81)	DEC/AZA plus Venetoclax	3(1–7)	ORR: 18/32,56.3%; CR/CRi:14/32,43.8%; PR:4/32, 13%	8(1–24)	NA
Masarova et al. [27] USA, 2021	Retrospective study	*N* = 18 MPN-BP/post-MPN AML	19(61)	69(46–80)	DEC + Venetoclax	5(1–14)	ORR:5/18, 27.8%; CR/CRi:4/18, 22.2%; PR: 1/18, 5.6%	NA	NA
King et al. [28] USA, 2021	Retrospective study	*N* = 27 MPN-AP: 6 MPN-BP: 21	18(66.7%)	72(55–82)	AZA/DEC + Venetoclax	2(1–15)	ORR:14/24, 58.3%; CR:10/24, 41.7%; PR: 4/24, 16.7%	NA	NA
Yoon et al. [29] USA, 2021	Retrospective study	*N* = 39 MPN-BP:39	7(43.8)	HMA/Ven: 76(52–82)	DEC/AZA plus Venetoclax	NA	ORR:3/7, 42.9%; CR/CRi:3/7, 42.9%	5	Infection/fever(57.1%), haemorrhage(28.6%)
Zhou et al. [10] USA, 2021	Retrospective study	*N* = 42 MPN-AP: 14 MPN-BP: 16 MPN-HR: 12	16(61.5)	70.3(48.7–82.8)	DEC plus RUX	5.5(1–56)	NA	21(4.5–NR)	Bacterial infection, viral infection, fungal infection, arterial thrombus, venous thrombus, haemorrhage

*Note:* DEC: decitabine; AZA: azacytidine; HMAs: hypomethylating agents; GO: gemtuzumab ozogamicin; RUX: ruxolitinib; MOS: median overall survival; ORR: overall response rate; CR: complete remission; CRi: CR with incomplete count recovery; PR: partial remission; MLFS: morphological leukaemia free state; NR: not reach; NA: not available.

**Table 2.** Overview of the efficacy and safety of HMAs monotherapy in Philadelphia negative MPN-AP/BP.

Source (author/ country)	Study type	Population	Number of men, n (%)	Median age (year, range)	Regimen	Number of cycles, median (range)	Efficacy	Median OS, months (range)	Adverse events
Thepot et al. [11] France, 2010	Retrospective study	*N* = 26 Post-MPN AML	NA	NA	AZA	6(1–28)	ORR: 10/26,38.5%, CR/CRi:4/26, 15.4%; PR: 6/26, 23.1%	8	Not reported in post-MPN AML patients
Andriani et al. [30] Italy, 2014	Retrospective study	*N* = 19 MPN-BP	15(78.9%)	71.3(IQR: 64.5–77.7)	AZA	NA	ORR: 6/19, 31.6%; CR/CRi:5/19,26.3; PR:1/19, 5.3%	9.9(6.7–13.1)	grade 3/4 haematological AEs (21%), Non-haematological AEs: 1 episode of fever of unknown origin, 1 pulmonary fungal infection, 1 respiratory failure
Badar et al. [31] USA, 2014	Retrospective study	*N* = 43 MPN-AP: 19 MPN-BP: 24	31(72.1%)	MPN-AP: 66(50–77) MPN-BP: 63(22–82)	DEC	MPN-AP: 3(1–31) MPN-BP: 2(1–15)	MPN-AP: ORR: 6/19, 32% MPN-BP: ORR:6/24, 25% CR/CRi:5/24, 21% PR:1/24, 4%	MPN-AP:14.8 MPN-BP: 6.9	NA
Badar et al. [32] USA, 2015	Retrospective study	*N* = 45 MPN-AP 13 MPN-BP 21 DIPSS plus high risk PMF 11	32(71.1)	MPN-AP: 63(50–81) MPN-BP: 64(45–82) DIPSS plus high risk PMF: 67(55–77)	DEC(*n* = 24,12AP,12BP)	MPN-AP: 2(1–6) MPN-BP: 2(1–13)	MPN-AP: ORR:1/45, 8% MPN-BP: ORR:9/21, 41.7%	MPN-AP:9.7 MPN-BP:6.9 DIPSS plus high risk PMF:2.7	NA
Andriani et al. [33] Italy, 2016	Retrospective study	*N* = 16 MPN-BP	12(75%)	63.5(51–81)	AZA	NA	ORR: 7/16, 43.8%; CR/CRi:4/16, 25%; PR: 3/16, 18.8%	8.1(1–49.5)	NA
Chihara et al. [34] USA, 2016	Retrospective study	*N* = 273(total) Post-MPN AML	139 (50.9%)	67(28–92)	HMAs(*N* = 97) HDAC(*N* = 71) LDAC(*N* = 50)	NA	HMAs: ORR:29/97,30%; CR/CRi:29/97,30%	7.8	NA
Andriani et al. [35] Italy, 2017	Retrospective study	*N* = 32 MPN-BP	21(65.6%)	70.4(IQR:64.2–77.1)	AZA	NA	ORR:12/32,37.5%; CR/CRi:8/32, 25%; PR:4/32,15.6%	11.7(3.5–19.8)	NA
Dumas et al. [36] France, 2017	Retrospective study	*N* = 32 Post-MPN AML	17(53.1%)	70.5(61.2–83.3)	AZA(*n* = 18)	NA	ORR:3/18,16.7%	8.4(4.8–13.2)	NA
Venton et al. [37] France, 2017	Retrospective study	*N* = 73 Post-MPN AML	46(63%)	70(38–89)	AZA	NA	HMAs(*n* = 11): ORR:6/11,54.5%	7.9(2.3–24.3)	NA
Lancman et al. [22] USA, 2018	Retrospective study	*N* = 57 MPN-BP	33(58)	68(40–92)	AZA	2.5(1–32)	HMAs with or without additional therapies: CR:9/57,15%	6.7	NA
Mollard et al. [38] France, 2018	Retrospective study	*N* = 24 MPN-AP:10 MPN-BP:14	14(58.3%)	72.3(57.7–86.7)	AZA	1–18	ORR:12/24,50%; CR/CRi:8/24, 33%; PR:4/24, 17%	9	NA
Tefferi et al. [39] USA, 2018	Retrospective study	*N* = 26 MPN-BP	NA	NA	HMAs	NA	ORR:1/26,4%; CR:1/26, 4%	5.5	NA
Andriani et al. [40] Italy, 2019	Retrospective study	*N* = 39 MPN-AP:16 MPN-BP:23	NA	69.9(63.9‐76.9)	AZA	6(3–9)	ORR: 15/39,38.5%; CR/CRi:8/39,20.5% PR:7/39,18%,	13.5(8.2–18.7)	NA
Yoon et al. [29] USA, 2021	Retrospective study	*N* = 39 MPN-BP:39	7(43.8)	HMAs only: 72(62–83)	DEC/AZA (*n* = 9)	NA	ORR:1/9,11.1%, CR:1/9, 11.1%	1	Infection/fever (77.8%), haemorrhage (11.1%)
Zhou et al. [10] USA, 2021	Retrospective study	*N* = 42 MPN-AP: 14 MPN-BP: 16 MPN-HR: 12	16(61.5)	70.3(48.7–82.8)	DEC (*n* = 18, 7AP, 11BP)	5.5(1–56)	NA	12.9(6.1–NR)	Bacterial infection, fungal infection, arterial thrombus, venous thrombus, haemorrhage

DEC: Decitabine, AZA: Azacitidine, HMAs: Hypomethylating agents; MOS: Median overall survival, ORR: Overall Response Rate, CR: Complete Remission, CRi: CR with incomplete count recovery, PR: Partial Remission; HI: Haematologic Improvement; PD: Progress Disease; HDAC: High Dose Ara-c; LDAC: Low Dose Ara-c; NR: not reach; NA: not available.

### Response to HMAs plus venetoclax vs HMAs alone in MPN-AP/BP

**Figure 2.**
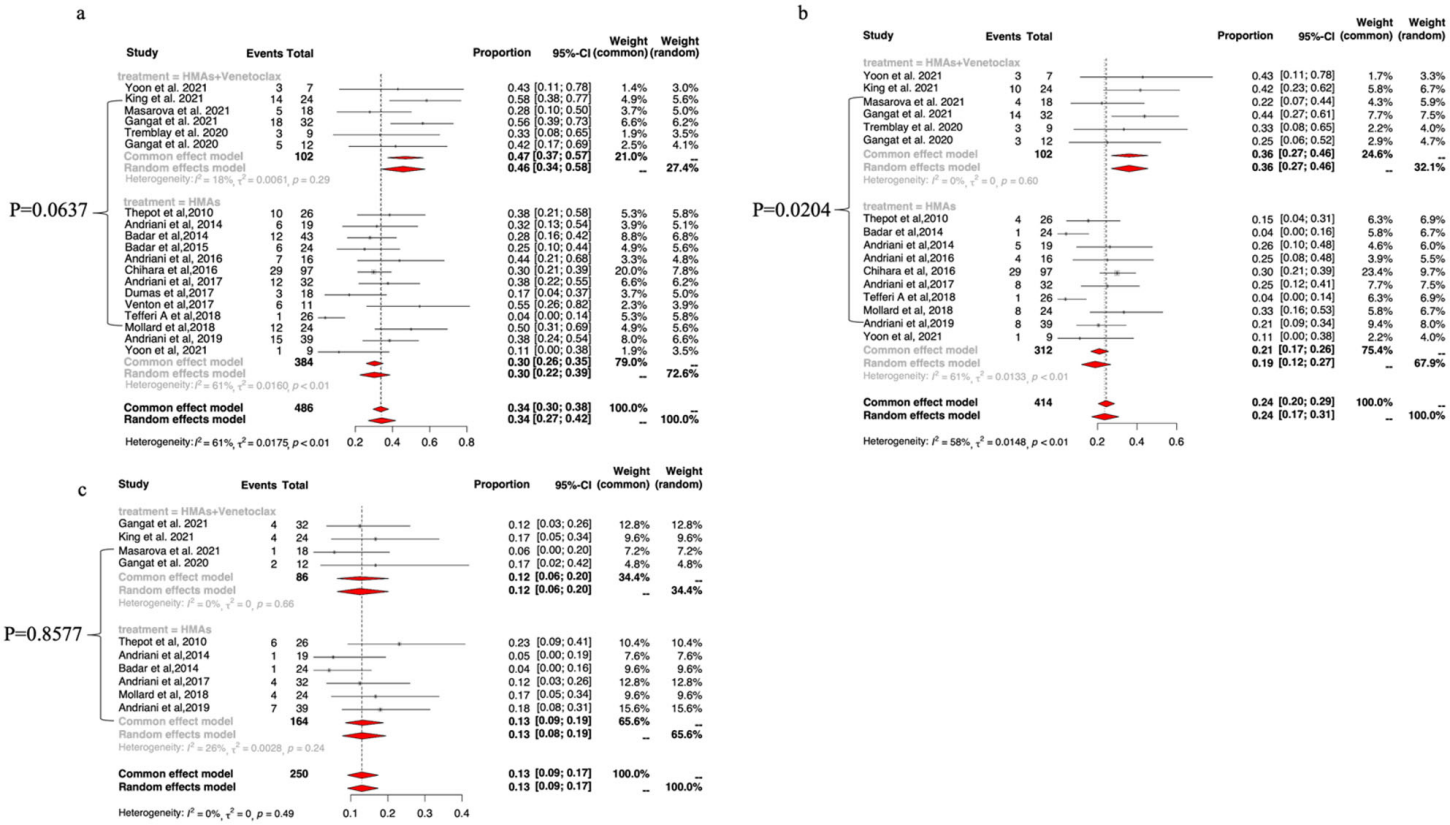
The pooled ORR (a), CR/CRi (b) and PR (c) rates of HMAs plus venetoclax vs HMAs alone in patients with MPN-AP/BP.

The pooled ORR was higher with HMAs plus venetoclax (47%, 95% CI: 37–57%; I^2^=18%) than with HMA monotherapy (30%, 95% CI: 22–39%; I^2^=61%), but the difference was not statistically significant in meta-regression analysis (*p* = .0637; Figure 2(a)). CR/CRi rates were reported by six studies of HMAs plus venetoclax therapy. The pooled CR/CRi rate was 36% (95% CI: 27–46%; I^2^=0%) for HMAs plus venetoclax and 19% (95% CI: 12–27%; I^2^=61%) for HMA monotherapy, with the difference reaching statistical significance in meta-regression analysis (*p* = .0204; Figure 2(b)). PR rates were reported by four studies on HMAs plus venetoclax combination therapy. The pooled PR rate was 12% (95% CI: 6–20%; I^2^=0%) for HMAs plus venetoclax and 13% (95% CI: 9–19%; I^2^=26%) for HMA monotherapy; the difference was not statistically significant in meta-regression analysis (*p* = .8577; Figure 2(c)).

A pre-specified subgroup analysis of patients with MPN-BP revealed that the pooled ORR and PR rates of HMAs plus venetoclax vs HMAs alone did not reach a statistically significant difference in meta-regression analysis (*p* = .0667 and *p* = .6190, respectively, Supplemental Figure 4 C a/c). The pooled CR/CRi rate was 37% (95% CI: 27–48%; I^2^=33%) for HMAs plus venetoclax and 19% (95% CI: 12–27%; I^2^=58%) for HMAs alone, which reached statistically significant difference in meta-regression analysis (*p* = .0286; Supplemental Figure 4C b). The data on treatment response in the enrolled studies was insufficient to conduct subgroup meta-analysis in MPN-AP patients.

### Response to HMAs plus ruxolitinib vs HMAs alone in MPN-AP/BP

**Figure 3.**
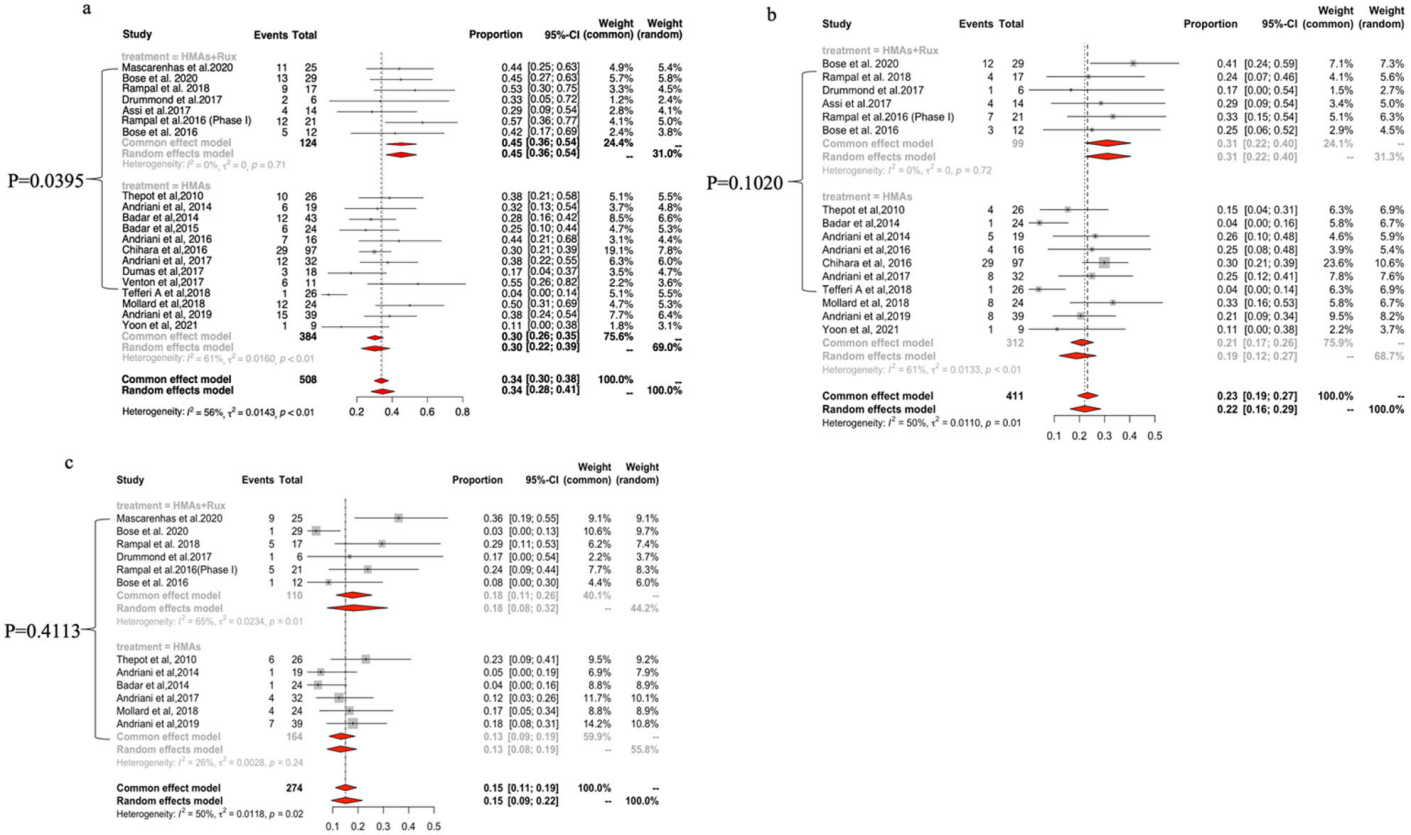
The pooled ORR (a), CR/CRi (b) and PR (c) rates of HMAs plus ruxolitinib (Rux) vs HMAs monotherapy in patients with MPN-AP/BP.

The ORR of pooled studies was 45% (95% CI: 36–54%; I^2^=0%) for HMAs plus ruxolitinib and 30% (95% CI: 22–39%; I^2^=61%) for HMAs alone, which reached a statistically significant difference in meta-regression analysis (*p* = .0395; Figure 3(a)). The pooled CR/CRi rate was 31% (95% CI: 22–40%; I^2^=0%) for HMAs plus ruxolitinib and 19% (95% CI: 12–27%; I^2^=61%) for HMA monotherapy, but there was no statistically significant difference in meta-regression analysis (*p* = .1020; Figure 3(b)). The pooled PR rate was 18% (95% CI: 8–32%; I^2^=65%) for HMAs plus ruxolitinib and 13% (95% CI: 9–19%; I^2^=26%) for HMAs monotherapy, with the difference not reaching a statistical significance in meta-regression analysis (*p* = .4113; Figure 3(c)).

A pre-specified subgroup analysis in patients with MPN-AP revealed a pooled ORR of 56% (95% CI: 32–79%; I^2^ = 0%) for HMAs plus ruxolitinib and 20% (95% CI: 3–47%; I^2^ = 63%) for HMAs alone, which reached a statistically significant difference in meta-regression analysis (*p* = .0464; Supplemental Figure 4A). With respect to MPN-BP, the pooled ORR, CR/CRi and PR rates for HMAs plus ruxolitinib did not show statistically significant differences in meta-regression analysis when compared with HMA monotherapy (Supplemental Figure 4B).

### Response to HMAs plus venetoclax vs HMAs plus ruxolitinib in MPN-AP/BP

We conducted subgroup and meta-regression analyses of the efficacy of HMAs plus venetoclax vs HMAs plus ruxolitinib. ORR, CRi and PR rates were similar in studies reporting treatment with HMAs plus venetoclax vs HMAs plus ruxolitinib [47% (95% CI: 37–57%) vs 45% (95% CI: 36–54%), *p* = .7872, 16% (95% CI: 9–26%) vs 23% (95% CI: 16–31%), *p* = .2879 and 12% (95% CI: 6–20%) vs 18% (95% CI: 8–32%), *p* = .4436, respectively; Supplemental Figures 5I, III, IV]. However, the pooled CR rate appeared to be more favourable in patients treated with HMAs plus venetoclax than HMAs plus ruxolitinib [22% (95% CI: 11–35%) vs 8% (95% CI: 3–15%), *p* = .0313; Supplemental Figure 5II].

### Response to azacytidine vs decitabine in MPN-AP/BP

For patients treated with azacytidine or decitabine monotherapy (Supplemental Figure 6), the ORR for all studies was 38% with significant heterogeneity among the studies (Cochran’s Q statistic = 21.1, *p* = .01; I^2^ = 57.3%). The ORR was reported by eight studies for azacytidine and two studies for decitabine. The pooled ORR did not show a statistically significantly difference (*p* = .1362) between azacytidine [42% (95% CI: 31–52%), I^2^=55%] and decitabine [27% (95% CI: 17–38%), I^2^=0%].

### Overall survival rates at one-year and two-years and rate of allo-HSCT

**Figure 4.**
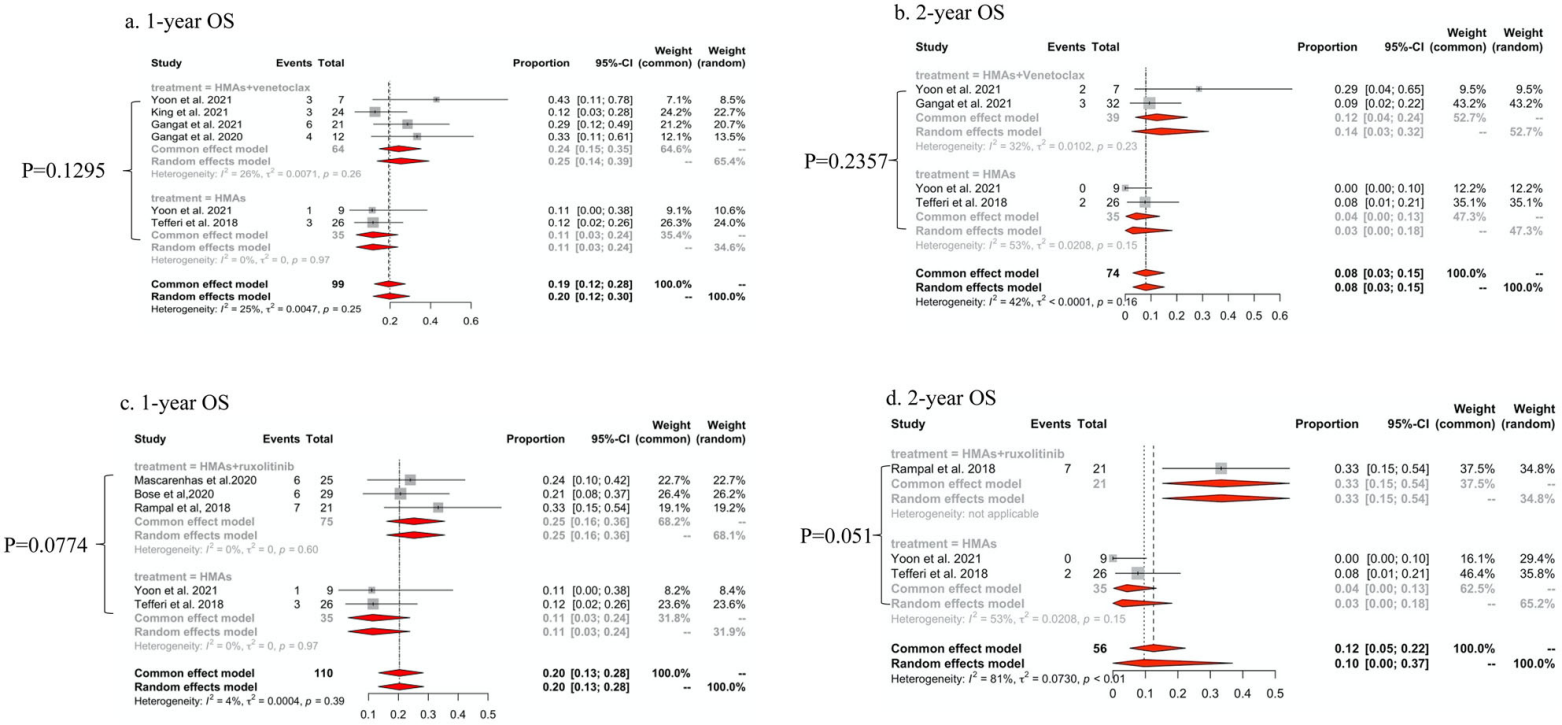
The pooled rates of one-year OS and two-year OS of HMAs plus venetoclax vs HMAs alone (a, b) and HMAs plus ruxolitinib vs HMAs alone (c, d) in patients with MPN-AP/BP.

Patients treated with HMAs plus venetoclax demonstrated a trend towards prolonged survival compared to those treated with HMAs alone [pooled one-year OS rates were 24% (95% CI: 15–35%; I^2^=26%) and 11% (95% CI: 3–24%; I^2^=0%) respectively; *p* = .1295, Figure 4(a); pooled two-year OS rates were 12% (95% CI: 4–24%; I^2^=32%) and 3% (95% CI: 0–18%; I^2^=53%) respectively; *p* = .2357; Figure 4(b)]. Similarly, patients treated with HMAs plus ruxolitinib showed a trend towards longer survival than HMAs alone [one-year OS rate: 25% (95%CI: 16–36%; I^2^=68.2%) and 11% (95% CI: 3–24%; I^2^=31.8%) respectively, *p* = .0774; two-year OS rate: 33% (95% CI: 15–54%; I^2^=34.8%) and 3% (95% CI: 0–18%; I^2^=65.2%), *p* = .051; Figure 4(c,d)].

Next, we summarized the proportion of patients with CR/Cri, who underwent allo-HSCT, in each treatment group (Supplemental Figure 7). There was a significant heterogeneity among the various studies, with a Cochran’s Q statistic of 33.45 (*p* < .001) and an I^2^ statistic of 72.9%. The rates of received allo-HSCT were 70% (95% CI: 34–95%; I^2^=62%), 25% (95% CI: 8–48%; I^2^=0%) and 54% (95% CI: 19–87%; I^2^=54%) for patients who received HMAs plus venetoclax, HMAs plus ruxolitinib and HMAs alone, respectively, with no statistically significant difference among groups in meta-regression analysis (Supplemental Figure 7).

### Adverse events

No data on haematologic AEs were reported by studies in patients treated with HMAs plus venetoclax or HMAs alone. However, three studies had reported haematological AEs in patients treated with HMAs plus ruxolitinib (Supplemental Figure 8). Pooled analysis of the three studies revealed that the most common haematologic AE was neutropenia (30%, 95% CI: 6–64%; I^2^=82%), followed by thrombocytopenia (25%, 95% CI: 0–70%; I^2^=90%), lymphopenia (19%, 95% CI: 6–38%; I^2^=NA) and anaemia (15%, 95% CI: 6–27%; I^2^=0%). The commonly reported non-haematologic AEs were infection/fever, haemorrhage and thrombus formation (Supplemental Figure 9). We were unable to compare AEs across different treatment groups owing to the significant heterogeneity and limited data availability. In addition, systematic evaluation of whether the AEs were treatment-related or secondary to any underlying comorbidity was not feasible.

### Sensitivity analysis

Separate sensitivity analyses for the pooled results of ORR, CR/CRi and PR with significant heterogeneity revealed that omitting any one study did not change the overall effect direction but did lead to a reduction in heterogeneity.

For ORR, the study by Tefferi et al. had the largest influence on heterogeneity [39], in the group examining the use of HMA monotherapy. Removal of this study changed the ORR by 3% (from 32% to 35%), and the higher degree of heterogeneity significantly decreased (Cochran’s Q statistic = 26.63, *p* = .0052; I^2^ = 58.7%). Furthermore, removal of this study led to a loss of heterogeneity in the subgroup analysis of studies examining the use of HMA monotherapy in MPN-BP patients (Cochran’s Q statistic = 8.08, *p* = .3255; I^2^ = 58.7%). Removal of this study also reduced the CR/CRi by 2% (from 19% to 21%) and led to a loss of heterogeneity among studies examining the use of HMA monotherapy (Cochran’s Q statistic = 14.26, *p* = .0753; I^2^ = 43.9%).

## Discussion

The outcomes of patients with MPN-AP/BP are generally poor, and therapy for this patient population represents a major unmet need. In the absence of direct comparisons of different treatment strategies due to a lack of randomized controlled trials in this area, it is not possible to suggest an optimal regimen. To the best of our knowledge, this is the first systematic review and meta-analysis on combination therapy vs monotherapy of HMAs in patients with MPN-AP/BP.

This meta-analysis summarizes the currently available evidence on the various therapy regimens in patients with MPN-AP/BP. The available evidence, albeit with low certainty and moderate risk of bias, suggests that the combination of HMAs and venetoclax is associated with a higher rate of CR/CRi than HMAs alone. HMAs plus ruxolitinib showed better ORR than HMA monotherapy, but subgroup analyses demonstrated a significantly lower CR rate than HMAs plus venetoclax. Patients treated with combination therapy showed a trend towards longer survival compared with HMAs alone. These results clearly support HMA-based combination therapy in in patients with MPN-AP/BP.

Data pertaining to efficacy in MPN-AP/BP patients treated with HMAs alone were inconsistent, and evidence of efficacy in comparison to HMAs plus venetoclax therapy remains insufficient. A retrospective study that enrolled 19 MPN-BP patients treated with azacytidine alone, reported an ORR of 26% [30]. However, another retrospective study conducted by Mollard et al. demonstrated an ORR of 50% in patients treated with azacytidine alone [38]. Only a small cohort study has demonstrated that MPN-BP patients receiving HMAs plus venetoclax achieved a higher CR rate compared to HMAs alone (25% vs 4%, *p* = .048) [41]. In the current analysis, HMAs plus venetoclax therapy was associated with improved efficacy when compared with HMA monotherapy, suggesting that combination therapy has an advantage over monotherapy and should be routinely recommended for MPN-AP/BP patients.

Rampal et al. demonstrated that combination treatment with HMAs and ruxolitinib was well tolerated by MPN-AP/BP patients, with an ORR of 53% [12]. In a multicentre, phase II clinical trial, a 44% ORR was achieved in 25 MPN-AP/BP patients treated with ruxolitinib in combination with decitabine, although response was not associated with improved survival [25]. However, it was unclear whether HMAs plus ruxolitinib therapy was more effective than using HMAs alone. In the meta-regression analysis of the present study, ORR was found to be significantly different between the patients treated with HMAs plus ruxolitinib and those treated with HMAs alone. Our results also revealed that HMAs plus ruxolitinib combination achieved a higher ORR than HMAs alone in patients with MPN-AP. However, it is important to note that only two previous studies have reported ORR in MPN-AP patients treated with HMAs plus ruxolitinib. Therefore, the effect of this combination in patients with MPN-AP needs further investigation.

Two independent, nonrandomized trials demonstrated acceptable safety and high efficacy of HMAs plus venetoclax therapy in AML patients, who were ineligible for intensive chemotherapy [42,43]. To further compare the efficacy of HMAs plus venetoclax and HMAs plus ruxolitinib regimens in patients with MPN-AP/BP, we performed a subgroup analysis which revealed that the venetoclax-based combination had significantly better efficacy compared to HMAs plus ruxolitinib (CR: 22% vs 8%, *p* = .0313). These results suggest the possibility of a significant association between HMAs plus venetoclax combination treatment and blast count reduction in MPN-AP/BP. Further randomized head-to-head trials are needed to confirm our findings.

Azacytidine and decitabine are widely used in clinical practice, and both have beneficial effects in unfit AML or higher-risk MDS patients [44]. However, only a few trials and retrospective studies have been conducted to directly compare the efficacy of azacytidine and decitabine in patients with MPN-AP/BP. Our meta-analysis included eight studies with 185 patients receiving azacytidine alone, and two studies with 67 patients receiving decitabine alone. The difference in the pooled ORR between azacytidine and decitabine was not statistically significant. Because all of the studies included in this meta-analysis were single-arm tests, there was a low certainty of evidence when comparing azacytidine and decitabine. Therefore, more head-to-head trials are needed in the future.

The goal of care for patients with MPN-AP/BP may still be haematopoietic cell transplantation (HCT) consolidation. Pooled analyses of OS rates in our study showed that patients treated with HMAs plus venetoclax or ruxolitinib tended to survive longer than patients treated with HMAs alone; however, the difference was not statistically significant. This may be explained by the fact that nearly half of the patients who responded to monotherapy with HMAs received allo-HSCT. However, only 25% of the patients who responded to HMAs plus ruxolitinib received transplantation, possibly due to the limited number of enrolled studies on combination therapy. Findings from recent studies corroborate our results that HMAs in combination with venetoclax prolong OS in secondary AML patients [45,46]. Based on the results of our study, the therapeutic option of HMAs combined with venetoclax increases the possibility of allo-HSCT and should be considered as a potential treatment bridge to HCT in a subset of patients.

There was significant heterogeneity in the present meta-analysis due to the small sample size, single-arm nature of most studies and the variety of reported treatment options. However, the heterogeneity did not significantly affect the results of the meta-analysis by sensitivity analyses after accounting for the 'leave-one-out’ method. In comparison to other studies, Tefferi et al. [39] found a lower ORR and CR rate with HMA monotherapy in MPN-AP/BP patients. This can be explained by the fact that the PR rate and number of cycles of HMA treatment were not reported in this study [39]. In addition, most patients (81%) of the cohort had an abnormal karyotype which has been validated as a poor prognostic factor. Excluding the study by Tefferi et al. [39], heterogeneity in the analysis of ORR or CR/CRi rate decreased significantly with HMA monotherapy. This indicates that response to treatment with HMAs alone can be assessed more accurately by strict selection of MPN-AP/BP patients.

### Limitations

This meta-analysis had several limitations. First, we had to rely on the response definitions provided by the researchers of the original studies. The degree of heterogeneity decreased but remained in our sensitivity analysis, which argues against a systematic effect of response definitions on our results. Second, since medical nomenclature has evolved over time, we cannot exclude the possibility that studies using terms likes ‘myeloproliferative disorders (MPD)’ instead of ‘myeloproliferative neoplasms or MPN’ might have been left out. However, the term ‘myeloproliferative disorders (MPD)’ has been officially renamed as ‘myeloproliferative neoplasms (MPN)’ since 2008, and HMAs combined with ruxolitinib or venetoclax have gained popularity in the treatment of unfit patients with MPN-AP/BP or AML only in recent years. Thus, it is unlikely that a more extensive search strategy would have changed the overall conclusions of this study.

## Conclusions

Our data reflect that combination therapy of HMAs with venetoclax is an effective and well-tolerated option in MPN-AP/BP patients, which is suitable for use both before and after HSCT. Our findings also support the routine use of HMAs plus ruxolitinib in MPN-AP patients. Randomized clinical trials and head-to-head studies of HMA-based combinations are warranted to provide evidence for optimal combination therapy.

## Supplementary Material

Supplemental Material

## Data Availability

All data generated or analysed during this study are included in this article and its supplementary information files. The data that support the findings of this study are available from the corresponding author upon reasonable request.
