# Supplementary material for: Efficacy and safety of combination therapies vs monotherapy of hypomethylating agents in accelerated or blast phase of Philadelphia negative myeloproliferative neoplasms: a systematic review and meta-analysis
Source: Ann Med. 2023 Jan 16;55(1):348–60. doi: 10.1080/07853890.2022.2164611 (PMC9848335; doi:10.1080/07853890.2022.2164611)

**Efficacy and Safety of Combination Therapies vs Monotherapy of Hypomethylating agents in Accelerated and Blast Phase of Philadelphia Negative Myeloproliferative Neoplasms: A Systematic Review and Meta-analysis**

Jia Chen^1,2,3^, Kefei Wang^1,2^, Zhijian Xiao^1,2,3^ and Zefeng Xu^1,2,3^

**Affiliations**

^1^State Key Laboratory of Experimental Hematology, National Clinical Research Center for Blood Diseases, Haihe Laboratory of Cell Ecosystem, Institute of Hematology and Blood Diseases Hospital, Chinese Academy of Medical Sciences & Peking Union Medical College, Tianjin 300020, China.

^2^Tianjin Institutes of Health Science, Tianjin 301600, China.

^3^MDS and MPN Centre, Institute of Hematology and Blood Diseases Hospital, Chinese Academy of Medical Sciences & Peking Union Medical College, Tianjin 300020, China.

**Supplemental information**

**Contents**

**Supplemental Table 1.** Pubmed Search strategy

**Supplemental Table 2.** Embase search strategy

**Supplemental table 3.** Web of science search strategy

**Supplemental table 4.** Cochrane library search strategy

**Supplemental figure 1.** MINORS Scale

**Supplemental figure 2.** Heatmap of MINORS Scale to Assess the Study Quality

**Supplemental figure 3.** Funnel Plots to Estimate Publication Bias

**Supplemental figure 4.** Random and common (fix) effects meta-analysis of studies examining the efficacy between combination therapy and HMAs monotherapy in patients with MPN-AP/BP. A: MPN-AP patients treated with HMAs plus ruxolitinib vs HMAs alone; B: MPN-BP patients treated with HMAs plus venetoclax vs HMAs alone: ORR(I), CR/CRi (II), PR (III); C: MPN-BP patients treated with HMAs plus ruxolitinib vs HMAs alone: ORR(I), CR/CRi (II), PR (III).

**Supplemental figure 5.** Random and common (fix) effects meta-analysis of studies examining the efficacy between HMAs plus venetoclax and HMAs plus ruxolitinib treatments. I: ORR, II: CR, III: CRi, IV: PR.

**Supplemental figure 6.** Random and common (fix) effects meta-analysis of studies examining the efficacy between azacytidine (AZA) and decitabine (DEC) treatments.

**Supplemental figure 7.** Random and common (fix) effects meta-analysis of patients with CR/CRi received allogeneic haematopoietic stem cell transplant (allo-HSCT).

**Supplemental figure 8.** Pooled results of haematologic adverse events among patients treated with HMAs plus ruxolitinib.

**Supplemental figure 9.** Pooled results on non-haematologic adverse events (AEs) of HMAs plus venetoclax, HMAs plus ruxolitinib, and HMAs alone.

**Supplemental table 1.** Pubmed search strategy

#1、(Myeloproliferative neoplasm*) [Mesh]

#2、(Myeloproliferative Disorder*) [All Fields]

#3、(Disorder*, Myeloproliferative) [All Fields]

#4、(((Myeloproliferative neoplasm*) [Mesh]) OR ((Myeloproliferative Disorder*) [All Fields])) OR ((Disorder*, Myeloproliferative) [All Fields])

#5、(Decitabine [Mesh]) OR (5-Aza-2'-deoxycytidine[All Fields]) OR (5 Aza 2' deoxycytidine[All Fields]) OR (5-AzadC[All Fields]) OR (AzadC Compound[All Fields]) OR (Compound, AzadC[All Fields]) OR (5AzadC[All Fields]) OR (2'-Deoxy-5-azacytidine[All Fields]) OR (2' Deoxy 5 azacytidine[All Fields]) OR (5-Azadeoxycytidine[All Fields]) OR (5 Azadeoxycytidine[All Fields]) OR (Dacogen[All Fields]) OR (5-Deoxyazacytidine[All Fields]) OR (5 Deoxyazacytidine[All Fields]) OR (NSC 127716[All Fields]) OR (NSC-127716[All Fields]) OR (NSC127716[All Fields]) OR (Decitabine Mesylate[All Fields]) OR (Mesylate, Decitabine[All Fields])

#6、(Azacitidine [Mesh]) OR (5-Azacytidine[All Fields]) OR (5 Azacytidine[All Fields]) OR (Azacytidine[All Fields]) OR (Vidaza[All Fields]) OR (NSC-102816[All Fields]) OR (NSC 102816[All Fields]) OR (NSC102816[All Fields])

#7、#5 or #6

#8、#4 and #7

**Supplemental table 2.** Embase search strategy

| #1. 'decitabine'/exp |
| --- |
| #2. '5-aza-2-deoxycytidine':ab,ti |
| #3. '5 aza 2 deoxycytidine':ab,ti |
| #4. '5-azadc':ab,ti |
| #5. 'azadc compound':ab,ti |
| #6. 'compound, azadc':ab,ti |
| #7. '5azadc':ab,ti |
| #8. '2-deoxy-5-azacytidine':ab,ti |
| #9. '2 deoxy 5 azacytidine':ab,ti |
| #10. '5-azadeoxycytidine':ab,ti |
| #11. '5 azadeoxycytidine':ab,ti |
| #12. 'dacogen':ab,ti |
| #13. '5-deoxyazacytidine':ab,ti |
| #14. '5 deoxyazacytidine':ab,ti |
| #15. 'nsc 127716':ab,ti |
| #16. 'nsc-127716':ab,ti |
| #17. 'nsc127716':ab,ti |
| #18. 'decitabine mesylate':ab,ti |
| #19. 'mesylate, decitabine':ab,ti |
| #20. #1 OR #2 OR #3 OR #4 OR #5 OR #6 OR #7 OR #8 OR #9 OR #10 OR #11 OR #12 OR #13 OR #14 OR #15 OR #16 OR #17 OR #18 OR #19 |
| #21. 'azacitidine'/exp |
| #22. '5-azacytidine':ab,ti |
| #23. '5 azacytidine':ab,ti |
| #24. 'azacytidine':ab,ti |
| #25. 'vidaza':ab,ti |
| #26. 'nsc-102816':ab,ti |
| #27. 'nsc 102816':ab,ti |
| #28. 'nsc102816':ab,ti |
| #29. #21 OR #22 OR #23 OR #24 OR #25 OR #26 OR #27 OR #28 |
| #30. 'myeloproliferative neoplasm*' |
| #31. 'myeloproliferative disorder*':ab,ti |
| #32. 'disorder*, myeloproliferative':ab,ti |
| #33. #30 OR #31 OR #32 |
| #34. #20 OR #29 |
| #35. #33 AND #34 |

**Supplemental table 3.** Web of science search strategy

#1 TS=(Decitabine OR 5-Aza-2'-deoxycytidine OR 5 Aza 2' deoxycytidine OR 5-AzadC OR AzadC Compound OR Compound, AzadC OR 5AzadC OR 2'-Deoxy-5-azacytidine OR 2' Deoxy 5 azacytidine OR 5-Azadeoxycytidine OR 5 Azadeoxycytidine OR Dacogen OR 5-Deoxyazacytidine OR 5 Deoxyazacytidine OR NSC 127716 OR NSC-127716 OR NSC127716 OR Decitabine Mesylate OR Mesylate, Decitabine)

#2 TS=(Azacytidine OR 5-Azacytidine OR 5 Azacytidine OR Azacytidine OR Vidaza OR NSC-102816 OR NSC 102816 OR NSC102816)

#3 TS=(Myeloproliferative neoplasm* OR Myeloproliferative Disorder* OR Disorder*, Myeloproliferative)

#4 #1 OR #2

#5. #4 and #5

**Supplemental table 4.** Cochrane library search strategy

#1 (decitabine):ti,ab,kw (Word variations have been searched)

#2 (Azacytidine):ti,ab,kw (Word variations have been searched)

#3 #1 or #2

#4 (Myeloproliferative neoplasm*):ti,ab,kw

#5 (Myeloproliferative Disorder*):ti,ab,kw

#6 (Disorder*, Myeloproliferative):ti,ab,kw

#7 #4 or #5 or #6

#8 #3 and #7

**Supplemental figure 1.** MINORS Scale.


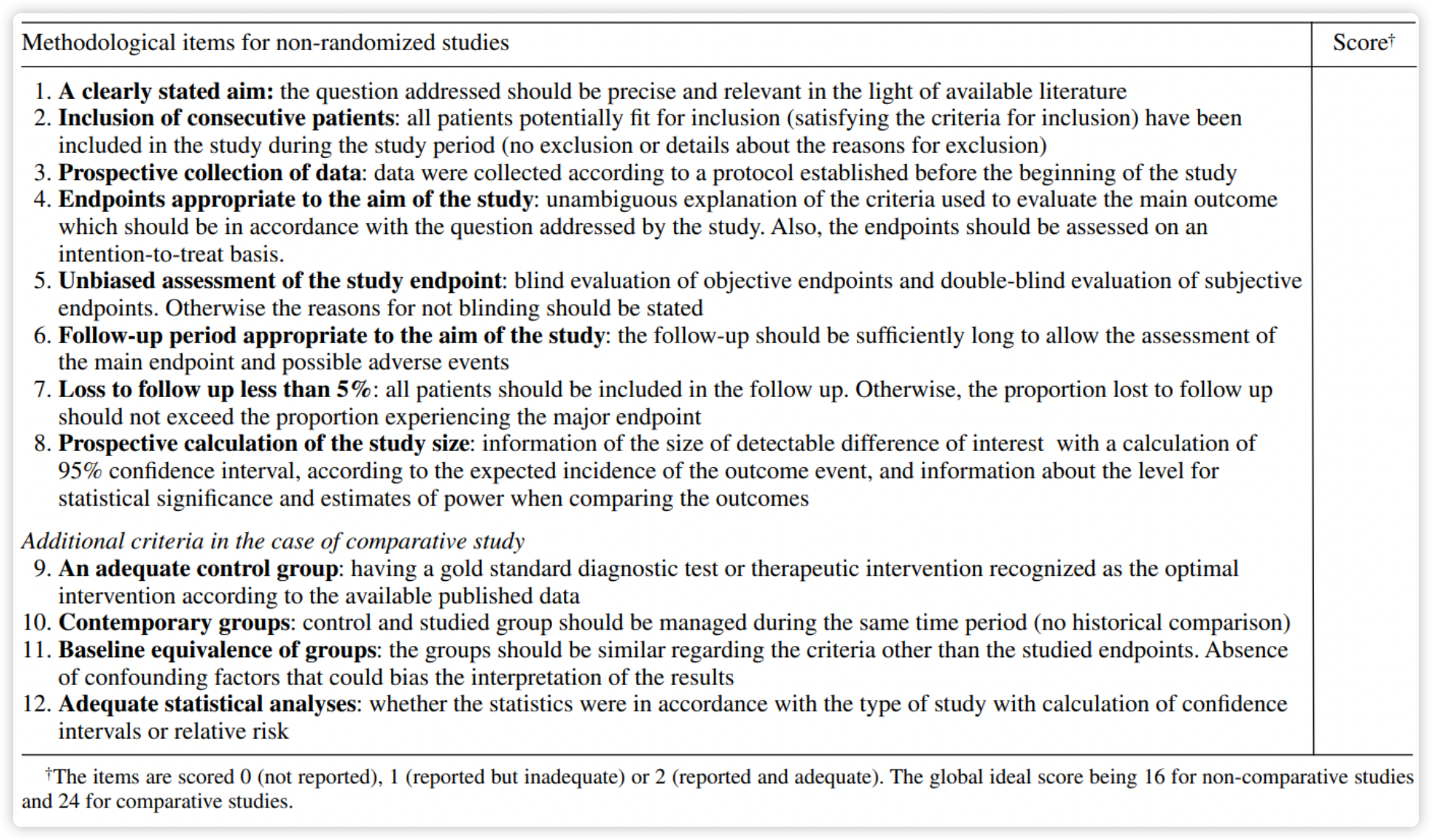


**Supplemental figure 2.** Heatmap of MINORS Scale to Assess the Study Quality.

**Supplemental figure 3.** Funnel Plots to Estimate Publication Bias

**
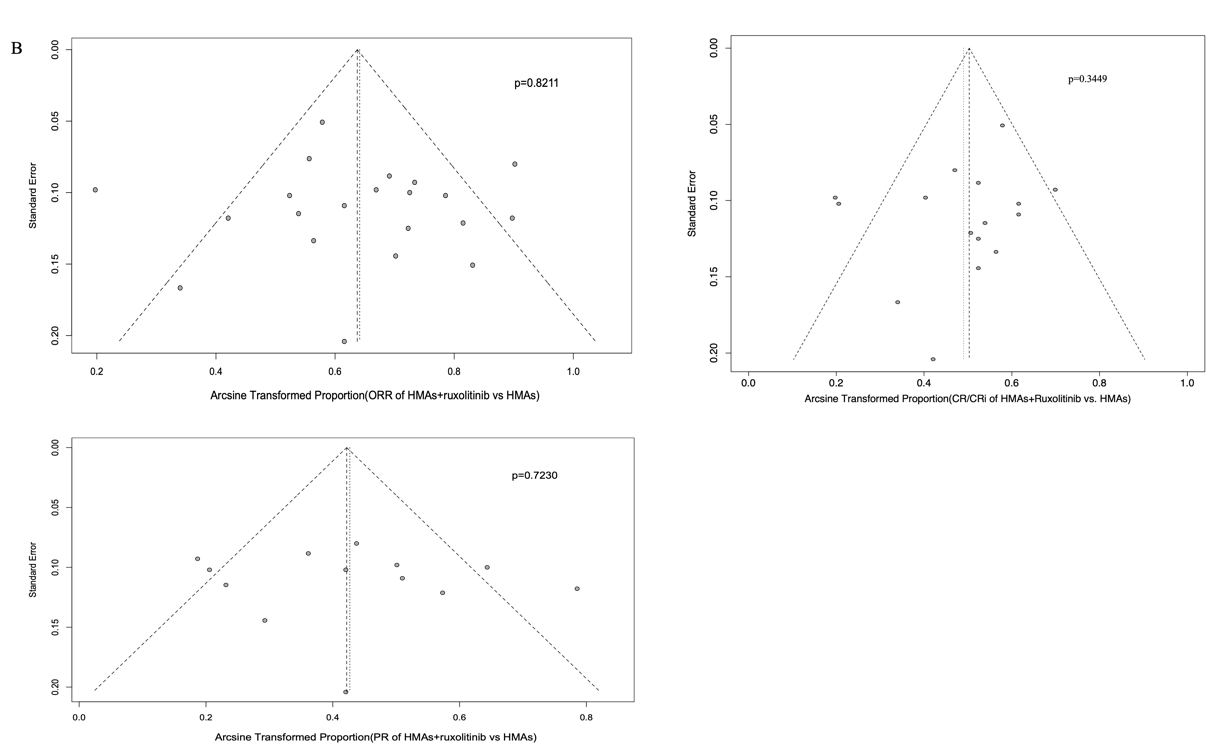
**

**Supplemental figure 4.** Random and common (fix) effects meta-analysis of studies examining the efficacy between combination therapy and HMAs monotherapy in patients with MPN-AP/BP. A: the ORR of MPN-AP patients treated with HMAs plus ruxolitinib vs HMAs alone; B: MPN-BP patients treated with HMAs plus ruxolitinib vs HMAs alone: ORR(I), CR/CRi (II), PR (III); C: MPN-BP patients treated with HMAs plus ruxolitinib vs HMAs alone: ORR(I), CR/CRi (II), PR (III).

A.

B.


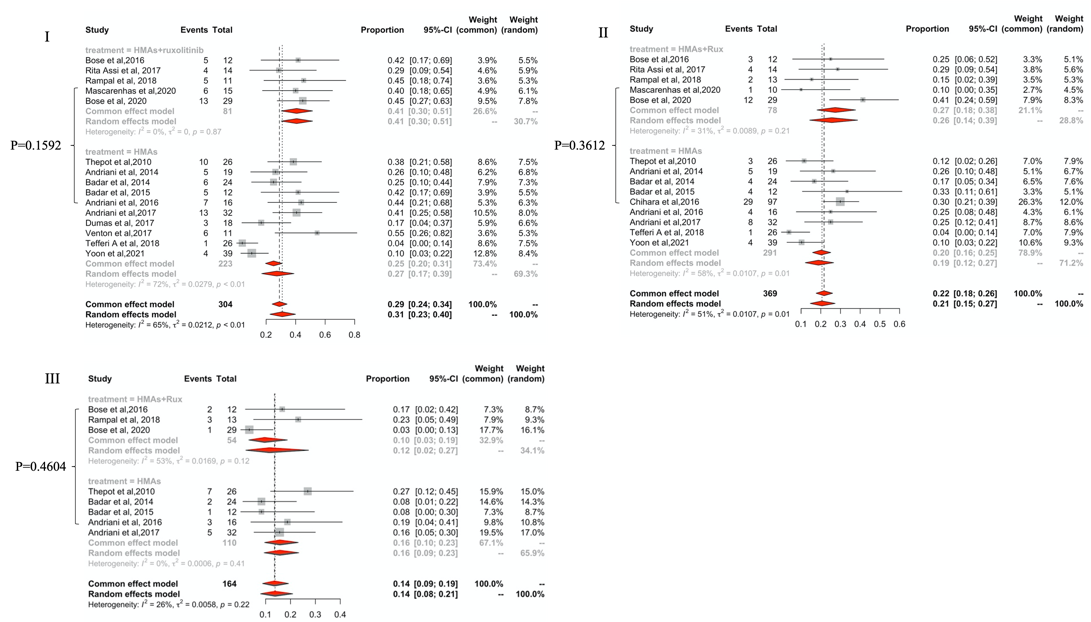


C.


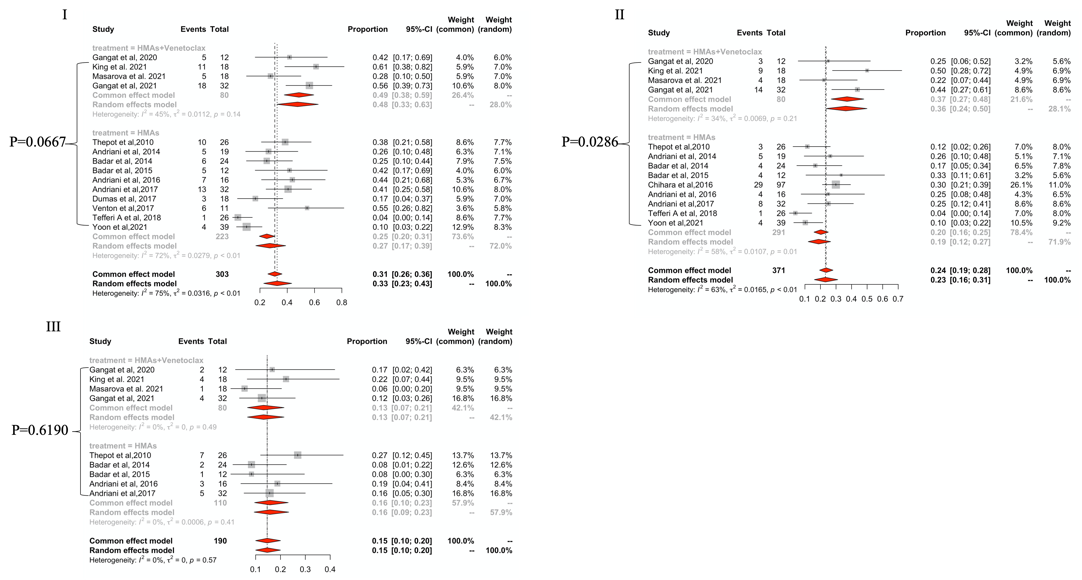


**Supplemental figure 5.** Random and common (fix) effects meta-analysis of studies examining the efficacy between HMAs plus venetoclax and HMAs plus ruxolitinib treatments. I: ORR, II: CR, III: CRi, IV: PR


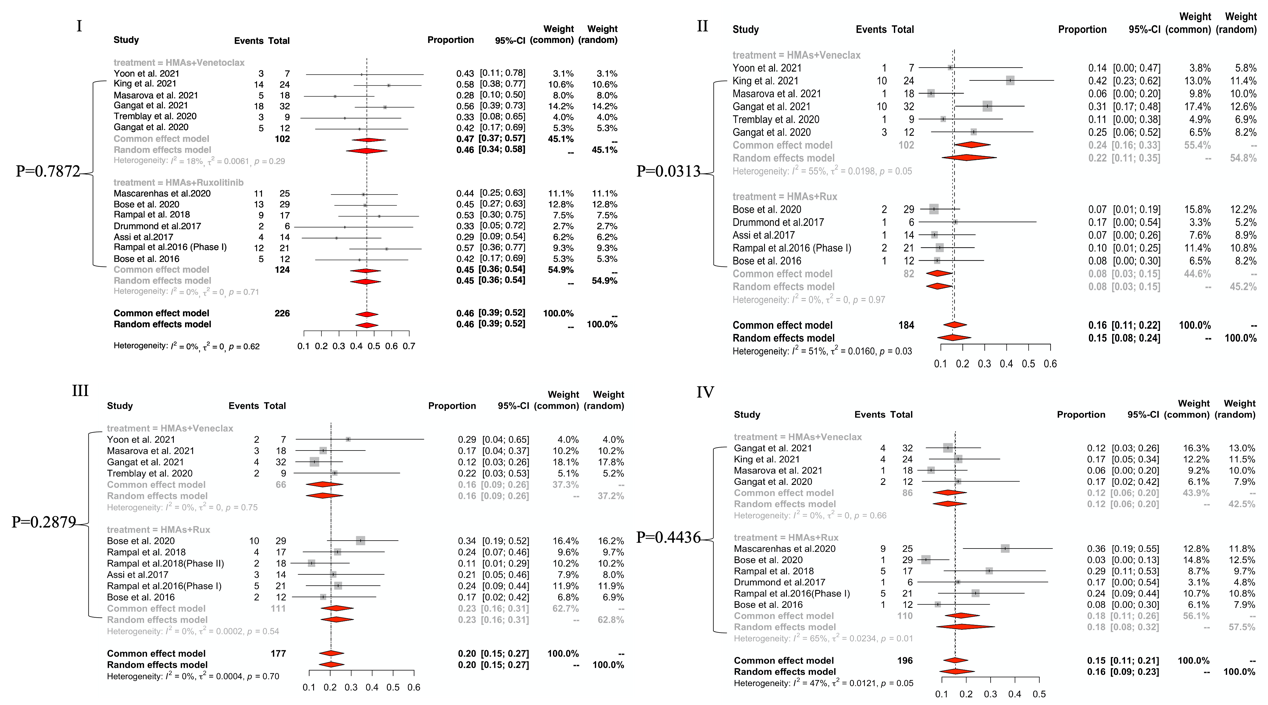


**Supplemental figure 6.** Random and common (fix) effects meta-analysis of studies examining the efficacy between azacytidine (AZA) and decitabine (DEC) treatments.

**Supplemental figure 7.** Random and common (fix) effects meta-analysis of patients with CR/CRi received allogeneic haematopoietic stem cell transplant (allo-HSCT).

rux: ruxolitinib; ven: venetoclax

**Supplemental figure 8.** Pooled results of haematologic adverse events among patients treated with HMAs plus ruxolitinib.


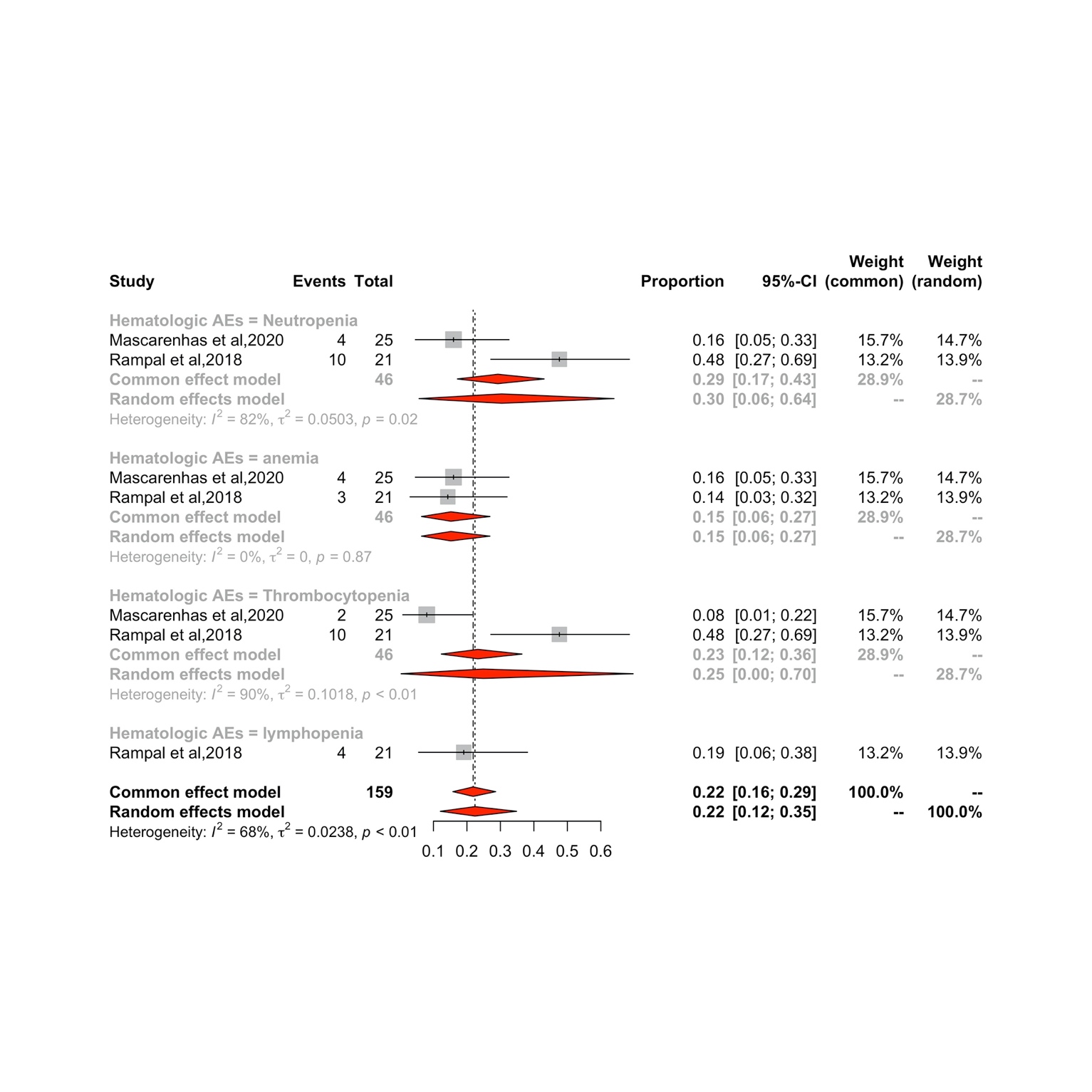


**Supplemental figure 9.** Pooled results on non-haematologic adverse events (AEs) of HMAs plus venetoclax, HMAs plus ruxolitinib, and HMAs alone.

1. **Infection/fever**


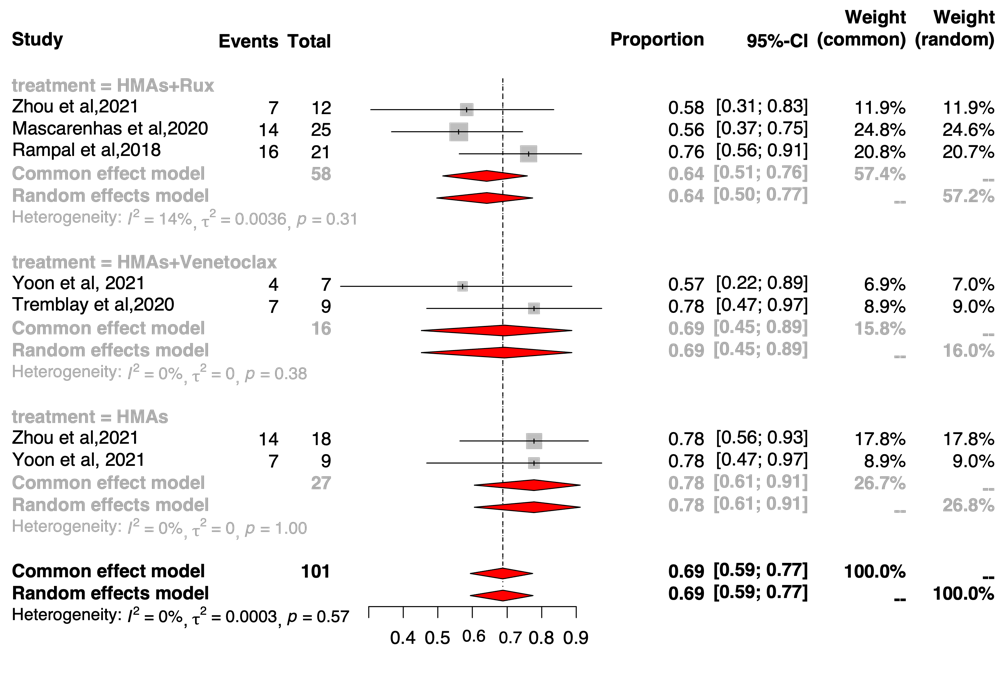


1. **Hemorrhage**


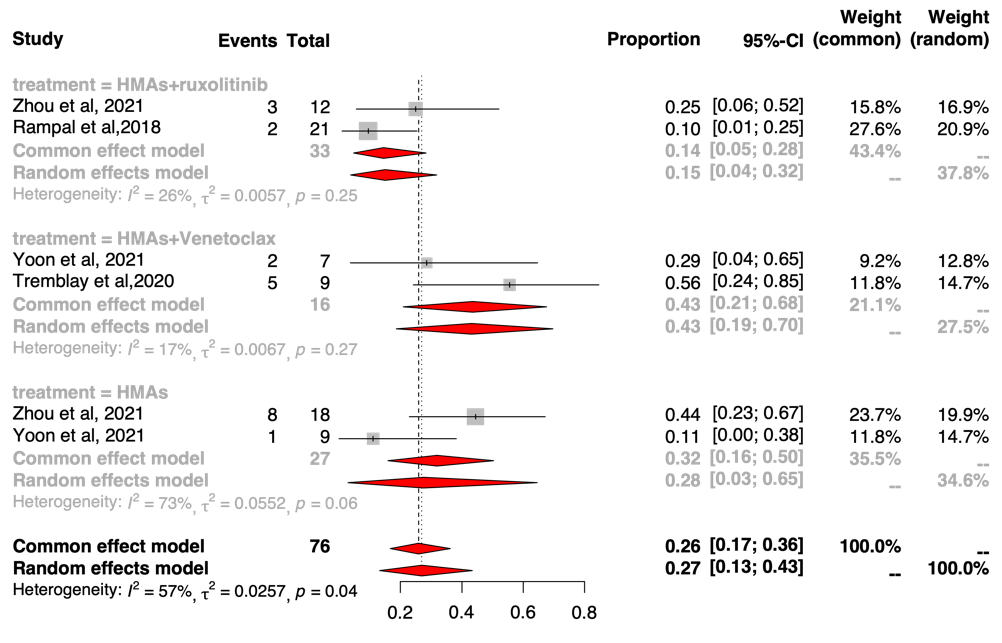


1. **Thrombus**


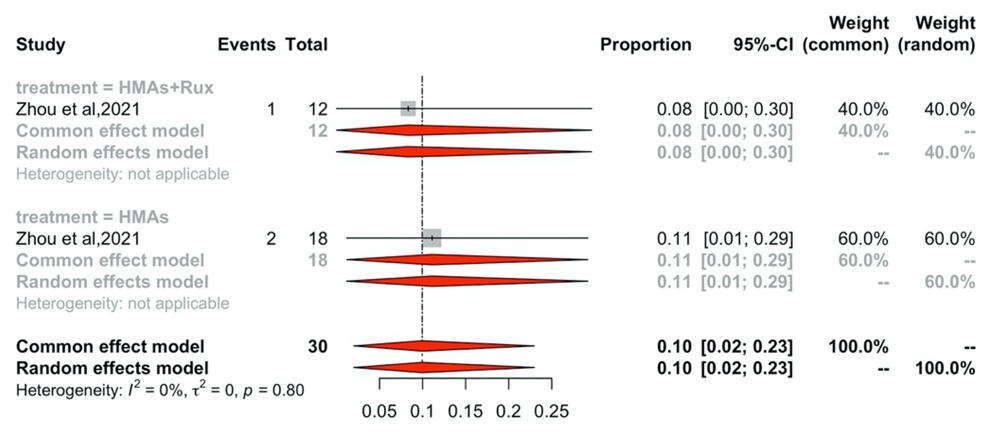

Supplement: Supplemental Material [file IANN_A_2164611_SM8337.docx]
